# Methods for detecting seasonal influenza epidemics using a school absenteeism surveillance system

**DOI:** 10.1186/s12889-019-7521-7

**Published:** 2019-09-05

**Authors:** Madeline A. Ward, Anu Stanley, Lorna E. Deeth, Rob Deardon, Zeny Feng, Lise A. Trotz-Williams

**Affiliations:** 1 Department of Mathematics and Statistics, University of Guelph, Stone Road, Guelph, N1G 2W1 Canada; 2 Department of Production Animal Health, University of Calgary, University Drive NW, Calgary, T2N 1N4 Canada; 3 Department of Mathematics and Statistics, University of Calgary, University Drive NW, Calgary, T2N 1N4 Canada; 4 Wellington-Dufferin-Guelph Public Health, Chancellors Way, Guelph, N1G 0E1 Canada

**Keywords:** Absenteeism surveillance system, Influenza, Seasonal logistic regression, Disease modelling, Epidemic detection

## Abstract

**Background:**

School absenteeism data have been collected daily by the public health unit in Wellington-Dufferin-Guelph, Ontario since 2008. To date, a threshold-based approach has been implemented to raise alerts for community-wide and within-school illness outbreaks. We investigate several statistical modelling approaches to using school absenteeism for influenza surveillance at the regional level, and compare their performances using two metrics.

**Methods:**

Daily absenteeism percentages from elementary and secondary schools, and report dates for influenza cases, were obtained from Wellington-Dufferin-Guelph Public Health. Several absenteeism data aggregations were explored, including using the average across all schools or only using schools of one type. A 10% absence threshold, exponentially weighted moving average model, logistic regression with and without seasonality terms, day of week indicators, and random intercepts for school year, and generalized estimating equations were used as epidemic detection methods for seasonal influenza. In the regression models, absenteeism data with various lags were used as predictor variables, and missing values in the datasets used for parameter estimation were handled either by deletion or linear interpolation. The epidemic detection methods were compared using a false alarm rate (FAR) as well as a metric for alarm timeliness.

**Results:**

All model-based epidemic detection methods were found to decrease the FAR when compared to the 10% absence threshold. Regression models outperformed the exponentially weighted moving average model and including seasonality terms and a random intercept for school year generally resulted in fewer false alarms. The best-performing model, a seasonal logistic regression model with random intercept for school year and a day of week indicator where parameters were estimated using absenteeism data that had missing values linearly interpolated, produced a FAR of 0.299, compared to the pre-existing threshold method which at best gave a FAR of 0.827.

**Conclusions:**

School absenteeism can be a useful tool for alerting public health to upcoming influenza epidemics in Wellington-Dufferin-Guelph. Logistic regression with seasonality terms and a random intercept for school year was effective at maximizing true alarms while minimizing false alarms on historical data from this region.

## Background

Influenza is one of the leading causes of death in Canada, with seasonal influenza resulting in 6000 - 20,000 hospitalizations and an average of 11.3 deaths per 100,000 population each year [1, 2]. Early detection of the onset of a seasonal influenza epidemic at the community level is important so that appropriate public health intervention measures can be taken. For example, the World Health Organization suggests several behavioural interventions for preventing the spread of influenza A (pH1N1) such as staying at home when ill and hand-washing [3]. Public health units can increase communications of these messages if they receive warning sufficiently early in an influenza epidemic [3], which may mitigate the severity of the epidemic due to public awareness. Ideally, the timing of this messaging should occur close enough to influenza season so that the public feels there is cause to follow suggestions, but as early as possible to maximise the effectiveness of mitigation measures.

Syndromic surveillance uses non-traditional indicators, such as over-the-counter medication sales [4, 5], ambulance dispatch data [6–8], and emergency department data [9, 10] for early detection of outbreaks or epidemics. Indirect health-related indicators such as these have been found to improve timeliness (or sensitivity) of surveillance systems, often resulting in an epidemic being detected sooner than it would have been if only clinical data were monitored [11]. However, the non-specific nature of this type of data can also lead to an increase in alarms that are not related to the disease of interest, reflecting decreased specificity for the surveillance system [11]. Therefore, the challenge of syndromic surveillance lies in finding an epidemic detection method that can produce a manageable number of false positive alarms, while still remaining sensitive enough to provide public health units with warning far enough in advance of the reporting of laboratory-confirmed cases to be useful.

### Influenza surveillance with school absenteeism

School absenteeism surveillance is of particular importance to public health because children aged five to fifteen years have been found to have the highest rates of influenza infection [12], and children under eighteen years old are the most likely family members to transmit influenza to the home [13]. Since most children spend a significant part of their time at school, schools likely play a significant role in spreading influenza to the wider community [13]. Furthermore, school absenteeism has been found to be significantly higher during influenza season than during the rest of the winter [14].

Several studies have used different epidemic detection techniques within influenza surveillance systems using school absenteeism data. These studies are often interested in either raising an alarm for the beginning of a seasonal influenza outbreak or epidemic, or measuring correlation between absenteeism and influenza. The studies that attempt to detect the start of an outbreak generally use techniques from one of three categories: thresholds (either fixed or individualized), models adapted from techniques traditionally used in statistical process control, and regression models.

In the first category, a study conducted in Quebec during the 2009 pH1N1 pandemic found the 10% threshold across all schools failed to detect outbreaks early enough for an intervention to be executed, either at the school-level or for the surrounding community [15]. The study also found that during an early wave of the pandemic, only around one third of schools met the absenteeism threshold, despite it being unlikely that none of the remaining schools had experienced an outbreak, indicating the 10% threshold may be too high for many schools and is not effective for early outbreak detection [15]. Mann et al. (2011) took a more individualized approach where an alarm could be raised when a school either surpassed 8% absenteeism or if absenteeism exceeded one standard deviation of the previous 30 day mean [16]. This study attempted to catch school-level outbreaks, however of the 89 schools that produced an alarm only nine were truly in the midst of an outbreak [16].

Examples of studies that use statistical process control techniques include Besculides et al. (2005), who used a cumulative sum (CUSUM) method to monitor absenteeism in New York City [17]. They examined three school years’ worth of absenteeism data and were able to detect changes in absenteeism for several community-wide epidemics of influenza-like illness [17]. However, they concluded that the absenteeism data still resulted in too much noise and did not ultimately recommend its implementation [17]. Similarly, Xu et al. (2017) applied CUSUM models to absenteeism from four schools in Tianjin, China [18]. They were able to detect 10 within-school outbreaks over the course of two school years, although they did not report the number of false alarms that were raised [18].

The final main modelling technique used for the early detection of seasonal influenza epidemics based on school absenteeism data is regression. A negative binomial regression model for predicting influenza epidemics using non-cause specific absenteeism in New York City was previously found not to be useful at giving advance notice [19]. However, Zhou et al. (2015) compared five statistical process control methods with linear and Poisson regression to see which one would provide the optimal alarm system for influenza where they use U.S. national syndromic (but not absenteeism) data. They reported that the regression models somewhat improved timeliness and sensitivity, especially for high influenza counts [20].

### Influenza surveillance in Wellington-Dufferin-Guelph

The Wellington-Dufferin-Guelph (WDG) region in Ontario, Canada covers two counties (Wellington, including the City of Guelph, and Dufferin). It encompasses approximately 4147 km^2^ and had a recorded population of 284,461 people in 2016 [21, 22]. At the time, the respective population densities of Dufferin and Wellington counties were 41.5 people/km^2^ and 83.7 people/km^2^ [21, 22]. Nearly half of the total population resided within the City of Guelph (in Wellington County), which covers 87 km^2^ and contains 53 elementary and secondary schools. Outside of Guelph, WDG is largely rural with six towns and nine townships.

The school absenteeism surveillance program at Wellington-Dufferin-Guelph Public Health (WDGPH) has utilized an on-line form to collect daily counts of students absent from schools in the WDG region since 2008. An absenteeism-based influenza surveillance program was piloted by WDGPH independently of the federal government in 2008 and became more established during the during the 2009–2010 pH1N1 epidemic. When absenteeism within a reporting school reaches 10%, WDGPH follows up with the school in question to investigate whether the increased absenteeism is related to illness and to advise on mitigating measures if it is. Cause of absenteeism is unavailable for most schools so WDGPH must use total all-cause absenteeism rather than symptomatic absenteeism with the threshold.

The 10% threshold used by several public health departments for absenteeism-based syndromic surveillance is an arbitrary threshold that is generally used for detection of any significant or widespread epidemic or outbreak within communities and schools. While some correlation has been noticed between the trends in absenteeism during an influenza season and trends in local school absenteeism both in WDG and elsewhere, there is no evidence that the 10% threshold is the best measure of unusual disease activity in a community or school. Further, the 10% threshold does not take into account the varying baseline absenteeism levels between different schools. For these reasons, there is a need to develop statistically sound approaches to using absenteeism data as a predictor of school or community disease activity.

This study evaluates several model-based alternatives to the 10% threshold for raising an epidemic alarm using school absenteeism data, with the goal of reducing these false alarms. The models include a statistical process control method, the exponentially weighted moving average (which approximates a Shewart chart or a CUSUM chart when different parameters are used [23]), as well as variations of logistic regression. In addition, two new metrics, false alarm rate (FAR) and accumulated days delay (ADD), are introduced to allow for epidemic detection method performance evaluation and comparison of the existing and proposed methods for detecting seasonal influenza epidemics using school absenteeism. The models used in the study were evaluated against influenza data from the community, as historically, there appeared to be no noticeable correlation between the number of cases of any other reportable disease in WDG and levels of school absenteeism. However, in most years there appeared to be a noticeable peak in absenteeism shortly before the peak in the incidence of reported cases of laboratory-confirmed seasonal influenza within the community.

## Methods

The goal of the methods discussed in this section was to detect a seasonal influenza epidemic within WDG earlier than it would be detected by waiting for reports of laboratory-confirmed influenza cases. For the purpose of this study, a seasonal influenza epidemic is defined as beginning when more than one case is observed within a seven-day period for the first time in any given influenza season. It is meant to reflect the increase in the occurrence of cases that occurs as the peak of an influenza season approaches, rather than the beginning of an influenza season.

Data from WDGPH were available from January 2008; however, at that time point, the 2007–2008 influenza season had already begun. Thus, the study period covered September 2008 to June 2018, not including the 2009–2010 school year. This year corresponded to the 2009 pH1N1 pandemic and was not used because, unlike in other years, the start of the epidemic preceded the start of the school year. Nine school years/ influenza seasons remained available to which the epidemic detection methods could be applied, although there were a limited number of elementary schools and no secondary schools reporting during the 2008–2009 school year as the program was still being piloted.

In the WDG region school years typically begin during the first week of September, and never before September 1^st^, so for consistency in analysis the school year was assumed to begin on September 1^st^ each year. All data cleaning, analysis, and visualization was performed in R version 3.5.0 [24].

### Data sources

#### Absenteeism data

Elementary and secondary schools within the WDG area are asked to report their absences to WDGPH each school day by 3:00 p.m. using an on-line form. The data obtained from WDGPH contained anonymized school identification numbers, the school population size, and the number of students absent for each day. Data from a limited number of schools also included the number of students absent due to illness, and specifically due to respiratory, gastrointestinal, and other symptoms; however, most schools did not provide this additional information. From the all-cause absenteeism, percentage absenteeism was calculated using total student population at the school as the denominator. Over the study period, 90 unique elementary schools and 14 unique secondary schools reported absenteeism data on at least one day. The number of schools reporting for a given day ranged from only one school to more than 40 schools in October-December 2010. The median number of schools that reported on school days was 13. No data were available for days when students were not required to attend school: weekends, statutory and school board holidays, and school breaks (for example, winter holidays, March break, and the summer holidays). In addition, schools that reported on fewer than five days throughout the study period were omitted from analysis. Extreme points where absenteeism was greater than 50%, and observations where elementary school population sizes were less than 45 or greater than 820 and secondary school population sizes were less than 443 or greater than 1902 (the smallest and largest consistently reported population sizes), were assumed to represent data entry errors and were therefore deleted from the dataset.

Differences in distribution between elementary and secondary school absenteeism were examined using the Mann-Whitney-Wilcoxon and Kolmogrov-Smirnov tests and, since both test results indicated a significant difference in the two distributions with a p-value <2.2×10^−16^, data from elementary and secondary schools were analyzed separately when fitting models. Different aggregation methods of absenteeism were explored, including using the average absenteeism of all schools of one type that reported on a day, using the average of the three most frequently reporting schools, and using data only from the most frequently reporting school. Sample means were calculated for each aggregation, along with 95% bootstrapped percentile intervals for the population (regional) means. Autocorrelation was accounted for in interval calculation; observations were sampled from blocks where block size was chosen from a geometric distribution with mean 20 and a sample size of 10000 [25], using the boot package in R [26, 27].

#### Influenza data

**Fig. 1:**
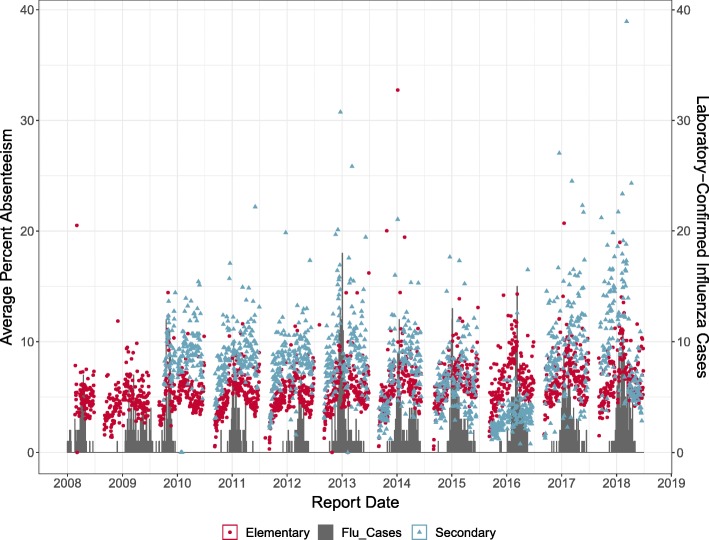
Influenza and absenteeism in WDGPH. Average absenteeism and laboratory-confirmed influenza cases (“Flu Cases”) reported to WDGPH for the WDG region from January 2008 to June 2018

The influenza dataset contained information about influenza cases in WDG that were laboratory-confirmed, and the dates on which WDGPH was notified of a case (Report Date) were used in analyses, with report date being chosen because this represented the date on which WDGPH first becomes aware of a case of influenza in the normal course of events. The dataset comprised usable data from nine influenza seasons, with seasonal epidemic start dates (reference dates) that ranged from late October to late January (Table 1, Fig. 1). The Spearman correlation coefficient was used to examine strength of relationship between averaged elementary and secondary school absenteeism and influenza case counts. Cross-correlation up to 15 lags was also calculated using the ccf function in R, with absenteeism and influenza counts ranked to approximate Spearman correlation.

**Table 1:** Reference dates representing the beginning of each seasonal influenza epidemic investigated in the study

School Year	Influenza Epidemic Reference Date
2008–2009	January 20^th^, 2009
2010–2011	December 14^th^, 2010
2011–2012	January 9^th^, 2012
2012–2013	October 26^th^, 2012
2013–2014	November 27^th^, 2013
2014–2015	December 8^th^, 2014
2015–2016	November 17^th^, 2015
2016–2017	December 15^th^, 2016
2017–2018	December 6^th^, 2017

The reference date was the report date for the second of two laboratory-confirmed influenza cases reported within seven days of each other for the first time in an influenza season

### Epidemic detection methods

Epidemic detection methods were applied to data prospectively. The first available year of data was used to train the models, and therefore was not used in model evaluation. Each school year was evaluated using models that had been trained on all data that temporally preceded that year. The school absenteeism featured missing values as described in the “Data sources” subsection. Both deletion and linear interpolation with the zoo package [28] were considered for treating these missing values. In addition, there were cases where a school reported more than once on a single date. We explored using either the maximum or the median of the reported values for that school to replace the absenteeism observations for those dates and schools. Every combination of the types of missing value and multiple entry handling was considered for each of the epidemic detection methods described in this section.

#### 10% threshold method

The method currently in use by WDGPH is a 10% absenteeism cut-off for raising an alarm for an outbreak (which may be related to influenza or a different disease). As it is currently used, whenever 10% or greater of the population at an individual school is absent, WDGPH follows up with that school to investigate possible illness. We explored raising an alarm for a region-wide epidemic with the 10% threshold method by using aggregated rather than individual school absenteeism data. Therefore an alarm was raised if absenteeism averaged across certain schools reached 10%. The threshold approach cannot be used with interpolated missing values as there are no parameters to be estimated, and it cannot incorporate any additional data or factors to aid in predicting the start of an epidemic.

#### Exponentially weighted moving average

Originally developed for use in econometrics, the exponentially weighted moving average (EWMA) and other statistical process control methods have been used in several influenza surveillance studies [29, 30]. The average of an epidemic-related variable is calculated, where observations in the past are given successively lower weights for determining the current test statistic [31]. Weights are represented by a parameter, *λ*, which can take values between 0 and 1 [31]. A value of *λ* close to 0 approximates a CUSUM chart, where all past observations are given equal weighting, while a *λ* value close to 1 approximates a Shewart chart, in which only the most recent observation is considered [23]. This gives the equation:

1 $$  z_{t} = \lambda x_{t} + (1 - \lambda)z_{t-1}  $$

where *x*_*t*_ is the value of the observed variable for day *t*, *z*_*t*_ is the EWMA statistic for day *t*, and *z*_*t*−1_ is the EWMA statistic for the day before day *t*. In this study, *x* was absenteeism aggregated in one of the ways described in the “Data sources” subsection, and either untransformed, log-transformed, or square root-transformed. Additionally, *t* indexes the days on which absenteeism data is available. For the first observed day, *z*_*t*−1_, or *z*_0_, was set to be the expected mean *μ*_0_ absenteeism when the process is “in-control” [32]. Here, *μ*_0_ was set as the mean absenteeism of the training years and thus was reset for each school year.

The variance of *z*_*t*_ can be found by expanding Eq. 1 to obtain:

2 $$  z_{t} = \lambda \sum_{j=0}^{t-1} (1 - \lambda)^{j} x_{t-j} + (1 - \lambda)^{t} z_{0},  $$

and taking the variance of Eq. 2. This gives:

$$\sigma^{2}_{z_{t}} = \sigma^{2} \left(\frac{\lambda}{2 - \lambda} \right) [1 - (1 - \lambda)^{2t}] $$

where *σ*^2^ is the in-control variance of *x*_*t*_. It was fixed at 1 for simplicity. The EWMA statistic is compared to a control limit and when *z*_*t*_ falls outside the bound of the control limit, an alarm is raised. Typically, both lower and upper control limits would be used. However, in the context of biosurveillance only the upper limit (UCL) is meaningful. The UCL is given by:

$$\text{UCL} = \mu_{0} + k \sigma^{2}_{z_{t}} $$

and an alarm was raised when *z*_*t*_> UCL. The parameters *λ* and *k* can be chosen theoretically to fix the error rate and make the UCL essentially equivalent to a one-sided 95% confidence limit. However, Buckeridge et al. (2005) found that in practice this results in unacceptable false alarm rates for most biosurveillance systems [33]. Therefore, in our study a range of values for the two parameters were used to fit the model, and the optimal values of *λ* and *k* were selected based on their performance on prediction with the evaluation metrics described in the “Evaluation metrics” subsection. Twenty values of *λ* between 0.05 and 1, and 20 values of *k* between 0.5 and 10 for untransformed data or 0.05 and 1 for transformed data were considered.

#### Logistic regression models

Distributed-lag regression models are used to analyse time series where the predictor variable is expected to correlate with a change of the response variable over a distributed period of time [34]. In the context of this study, it is likely that the first true influenza cases in the community preceded the first laboratory confirmed case each year, since not everyone who contracts an influenza infection will seek treatment from healthcare professionals [12]. In addition, there is a delay between the time when the first symptomatic case seeks health care and when results of the laboratory test for influenza are available. Because of this, it would be expected that an increase in absenteeism would be observed several days in advance of the report of any corresponding laboratory-confirmed cases to public health, and a distributed lag model would be appropriate to capture this phenomenon. Although EWMA models also take past observations of absenteeism into account, using a regression model allows for the inclusion of additional predictors.

Distributed-lag models use the value of a predictor variable for day *t* as well as for each day until *l* (the desired number of lags) days before day *t*. In this study, the outcome of interest was whether or not at least one case of (laboratory-confirmed) influenza would be reported to WDGPH on a given day, and thus logistic regression was used. Under this model the log-odds that at least one case occurs on day *t* is given by:

3 $$ {} \text{logit}(\rho_{t}) = \text{log}\left(\!\frac{\rho}{1-\rho}\!\right)\! =\! \beta_{0} + \beta_{1}x_{t}+ \beta_{2}x_{t-1}+... + \beta_{l+1}x_{t-l},  $$

where *ρ* is the probability of at least one case occurring on day *t*, *t*=1,…,*T*; *T* is the total number of days with absenteeism data available; *x*_*t*_ is the percentage of students absent on the given day, and *x*_*t*−*i*_ gives the percentage of students absent on the *i*^th^ day with absenteeism data available before day *t*. Although this model accounts for the potential delay between influenza circulation and reporting, it does not specifically take into account the seasonal pattern that influenza tends to follow. Thus, a second regression model that captures the seasonality pattern through the inclusion of trigonometric functions as covariates was considered [35]. The seasonal logistic regression model is given by:

4 $$  \begin{aligned} \text{logit}(\rho_{t}) &= \beta_{0} + \beta_{1}x_{t}+ \beta_{2}x_{t-1}+...\\ &\quad+ \beta_{l+1}x_{t-l} + \beta_{l+2} \text{sin}\left(\frac{2\pi t^{*}}{T^{*}}\right)\\ &\quad+ \beta_{l+3} \text{cos}\left(\frac{2\pi t^{*}}{T^{*}}\right), \end{aligned}  $$

where *t*^∗^ represents the calendar day of the year on which *x*_*t*_ was observed, and *T*^∗^ equals 365.25. When added together, the sine and cosine terms represent the harmonic motion of the response across the time axis, with a period of *T*^∗^ and amplitude of $\left (\beta _{l+2}^{2} + \beta _{l+3}^{2}\right)^{\frac {1}{2}}$ [35].

In addition to the lagged absenteeism predictors, versions of these models including an indicator variable for day of the week (DOW) were also considered, to account for possible weekly effects such as increased absenteeism each Monday or Friday.

The parameters for the above models were estimated using the glm function in R. For each of these regression models, as well as the mixed logistic regression and GEE models described below, lag lengths of 0 (only the current day’s absenteeism used) to 15 were considered. Alarms were raised when the predicted probability of at least one case being reported to WDGPH surpassed a defined threshold, *Θ*. Eleven possible thresholds between 0.1 and 0.6 were considered.

#### Mixed logistic regression models

To account for the effect of school years, models that included a random intercept for school year were also considered. Mixed regression models allow for intracorrelation among observations at a given measurement unit, such as multiple observations within an individual, a geographical location, or a time period [36]. For this study, including a random intercept for the observed absenteeism with a given school year acknowledges that absenteeisms, or their relationship to influenza may be correlated or similar within one year but vary over different years. Residual variance is divided into one component for the yearly level, and one component for the daily level [36].

Two mixed logistic regression models were examined in this study. The first added a random intercept for year to Eq. (3), giving:

5 $$ {} \text{logit}(\pi_{tj}) = \beta_{0} + \beta_{1}x_{tj}+ \beta_{2}x_{(t-1)j}+... + \beta_{l+1}x_{(t-l)j} + \gamma_{j}.  $$

The random component of the intercept is represented by *γ*_*j*_ and follows a normal distribution with mean 0 and variance *τ*^2^, and *j* indexes school year.

The second mixed model was an adaptation of Eq. (4):

6 $$ {} \begin{aligned} \text{logit}(\pi_{tj}) &= \beta_{0} + \beta_{1}x_{tj}+ \beta_{2}x_{(t-1)j}+...\\ &\quad+ \beta_{l+1}x_{(t-l)j} + \beta_{l+2}sin_{j}\left(\frac{2\pi t^{*}}{T^{*}}\right)\\ &\quad+ \beta_{l+3}cos_{j}\left(\frac{2\pi t^{*}}{T^{*}}\right) + \gamma_{j}, \end{aligned}  $$

with again *γ*_*j*_∼*N*(0,*τ*^2^). This model attempted to account for both the possible dependence of observations on school year as well as the seasonality of influenza. To fit the mixed models, we used the glmer command from the lme4 package in R [37]. Because the school year being modeled needed to be represented in the training dataset in order to estimate the intercept, data from September of the year of interest were included in the training data for each random intercept model.

#### Autoregressive GEE models

An alternative to generalized linear mixed models is to use a generalized estimating equation (GEE), which models data at the population level as opposed to the individual level [38]. School year was still included as a random effect, but a first order autoregressive correlation structure was also specified [38]. Due to the infectious nature of influenza, it is more likely there will be a new case if there has already been a case in the preceding days. Absenteeism follows a similar pattern, as illness spreads from child to child, so there are reasons to believe that observations which are closer together will be more highly correlated than those further apart and this can be modelled by a correlation structure. Two first order autoregressive GEEs were investigated in this study. The first is given by:

7 $$  \text{logit}(\mu_{tj}) = \beta_{0} + \beta_{1}x_{tj}+ \beta_{2}x_{(t-1)j}+... + \beta_{l+1}x_{(t-l)j}.  $$

Instead of estimating an individual probability of at least one case occurring, the model predicts the mean probability averaged across all observations with the same absenteeism values [39]. Therefore, *μ*_*j*_ represents the mean response for the population that has the same absenteeism pattern. The second model adds sine and cosine terms to create a seasonal variation on Eq. (7):

8 $${\kern7pt} \begin{aligned} \text{logit}(\mu_{tj}) &= \beta_{0} + \beta_{1}x_{tj}+ \beta_{2}x_{(t-1)j}+...\\ &\quad+ \beta_{l+1}x_{(t-l)j} + \beta_{l+2}sin_{j}\left(\frac{2\pi t^{*}}{T^{*}}\right)\\ &\quad+ \beta_{l+3}cos_{j}\left(\frac{2\pi t^{*}}{T^{*}}\right). \end{aligned}  $$

In both equations, *j* indexes school year, and *t*, *t*^∗^, and *T*^∗^ are as previously defined. The geeglm function from the geepack package in R was used to fit the GEE models [38].

### Evaluation metrics

For the purposes of this study, seasonal influenza epidemics were defined to begin when WDGPH was notified of two cases within a seven day period for the first time within an influenza season. Therefore these two cases could have been reported to WDGPH on the same day, or up to six days apart from each other. The reference day of the epidemic was the date of the second of these cases. Note that for the purposes of our analyses, the start of a seasonal influenza epidemic (i.e., the “reference day”) was defined differently from the usual definition of the start of an influenza season (the reporting of the first laboratory-confirmed case). Instead, the start of a seasonal influenza epidemic was defined as the report date of the second of two cases which had been reported to public health within seven days of each other. This was done in order to reflect the approaching peak of the season, as opposed to the relatively sporadic cases that often occur early in an influenza season.

Alarms were raised by EWMA models if the EWMA statistic, *z*_*t*_, surpassed the UCL, and by the regression models if the predicted probability of at least one laboratory-confirmed case occurring on day *t* surpassed the probability threshold *Θ*. Ideally, an alarm would be raised one to two weeks ahead of the start of a seasonal epidemic, so alarms were considered to be true if they occurred in the 15 calendar day period between the reference day of the epidemic and 14 days prior to the reference day, inclusive. An alarm raised prior to the reference day was considered to be false. Alarms raised between the day after the reference day and the final day of the school year were ignored.

Two metrics were used to optimize model parameters and evaluate the performance of the epidemic detection methods. The first, FAR, was calculated as:

9 $$ \text{FAR} = \left\{\begin{array}{ll} \frac{n_{f}}{n_{f} + 1}, & \,\,\text{if a true alarm was raised} \\ 1, & \,\,\text{if no true alarms were raised}, \end{array}\right.  $$

where *n*_*f*_ is the number of false alarms produced during that school year. The FAR produces a value between 0 and 1, where 0 would indicate that no false alarms and at least one true alarm were raised in a year. An FAR value of 1 or close to 1 would indicate that no true alarms were raised in a year, or that there was a large number of false alarms.

**Fig. 2:**
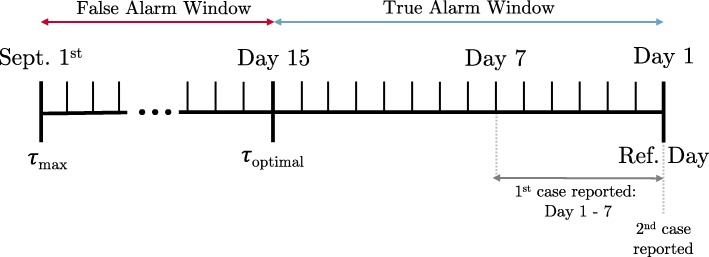
Evaluation metrics time-line. Illustration of terms used in the definitions of the evaluation metrics, ADD and FAR

The second metric was ADD. The ADD was used to give a sense of timeliness for true alarms. It was calculated as:

10 $${} \text{ADD} = \left\{\begin{array}{ll} \tau_{optimal} - \tau_{obs}, & \,\,\text{if a true alarm was raised} \\ \tau_{max}, & \,\,\text{if no true alarms were raised} \end{array}\right.  $$

where *τ*_*optimal*_ is 14 (or the ideal number of calendar days of advance notice before an epidemic reference day) and *τ*_*obs*_ is the number of calendar days before the epidemic reference day that the first true alarm raised for that season was declared. For example, if two true alarms were raised prior to the seasonal epidemic one year, one 12 days before the reference day and one 10 days prior, *τ*_*obs*_=12. In the event that no true alarms were raised, a large value was assigned to represent the system relying only on laboratory-confirmed cases. This value, *τ*_*max*_, was set to the number of days between the first day of the school year for which absenteeism data were available and the epidemic reference day, and so differed by year. See Fig. 2 for an illustration of the definitions of terms used in computing ADD.

Ideally, an epidemic detection method would have an ADD of 0, meaning a true alarm was observed 14 days before the epidemic reference day. An ADD of 14 would mean that the first true alarm was observed on the epidemic reference day. An ADD greater than 14 indicates no true alarm was observed that school year. Therefore any ADD value less than 14 indicates that the method was able to provide an alarm prior to when the epidemic would have been declared based on laboratory-confirmed influenza reports alone.

Metrics were calculated for each school year of every epidemic detection method, and then were averaged over all school years. For model-based methods, estimated parameters were chosen based on minimizing the average FAR. The models fitted with those optimized estimated parameters were then compared primarily by looking at which method could produce the lowest FAR, and among models that produced similar FARs, which method had the timeliest alarms as indicated by a low average ADD value.

## Results

### Preliminary data analysis

Mean daily all-cause absenteeism was 5.94%[95% CI = (5.58%, 6.31%)] for elementary schools and 7.76%[95% CI = (6.96%, 8.55%)] for secondary schools. Additional summary statistics for the different school aggregations considered in the epidemic detection methods are presented in Table 2.

**Table 2:** Summary of the different data aggregation types under consideration for use in epidemic detection methods

Data Type	Description	Mean	95% CI
Elementary (ES)			
ES-top	Daily absenteeism for the elementary school that reported the most days throughout the study period.	8.34%	(7.89%, 8.79%)
ES-3avg	Daily absenteeism averaged over the three elementary schools that reported the most days throughout the study period.	6.39%	(5.96%, 6.82%)
ES-allavg	Daily absenteeism averaged over all the elementary schools that reported.	5.94%	(5.58%, 6.31%)
Secondary (SS)			
SS-top	Daily absenteeism from the secondary school that reported the most days throughout the study period.	3.35%	(3.17%, 3.53%)
SS-3avg	Daily absenteeism averaged over the three secondary schools that reported the most days throughout the study period.	8.15%	(7.06%, 9.40%)
SS-allavg	Daily absenteeism averaged over all the secondary schools that reported.	7.76%	(6.96%, 8.55%)
ES.SS-allavg	Daily absenteeism averaged across all elementary and secondary schools that reported.	6.19%	(5.83%, 6.56%)

The sample means and bootstrapped (R = 10000) percentile 95% confidence intervals are given

Spearman correlation between influenza counts and average absenteeism was fairly weak, particularly for secondary schools. For elementary school absenteeism the correlation was 0.371, and for secondary school absenteeism it was 0.161. Cross-correlation was highest when elementary school absenteeism lagged behind influenza counts by six days (0.405) and when secondary school absenteeism was lagged by 11 days (0.181).

After removing missing and misreported values, the data aggregation type that had the most days of usable data was absenteeism averaged across all schools (ES.SS-allavg) with 1709 school days available out of the 3223 total calendar days in the study period. The average for all elementary schools (ES-allavg) had a similar number of school days available (1697), while the aggregation type with the fewest usable days was the top reporting secondary school (SS-top) with 1133 days available. This school did not begin reporting until the 2010–2011 school year and stopped reporting before the 2017–2018 school year. The top reporting elementary school (ES-top) had 1384 usable days.

### Epidemic detection methods

Using the maximum versus the median reported number of absences to replace entries when a school reported more than once within one day had little to no effect in most models, therefore only the results based on the maximum reported absence are presented in this section.

#### 10% threshold method

The 10% threshold method applied to the aggregated absenteeism data had limited ability to accurately identify epidemics ahead of laboratory confirmation. At best, this method produced an FAR of 0.661 with a corresponding ADD of 28.75 days when the average absenteeism across all secondary schools was used (Table 3). Allowing an alarm to be raised when any individual school reached 10% absenteeism generally resulted in lower ADD values, but very high FAR (0.827 at best). The most effective use of the threshold method gave true alarms for six out of eight evaluable school years, but up to 18 false alarms in five of the years. Although there was a high number of false alarms, the true alarms produced by this method were well-timed. Out of the six years where the start of a seasonal influenza epidemic was detected, only one of them had less then 10 days notice prior to the reference day.

**Table 3:** Evaluation metrics for the threshold-based epidemic detection methods where alarms are raised when absenteeism reaches 10%. Missing observations were not treated

Data Type	FAR	ADD
ES-top	0.799	37.75
ES-3avg	0.685	60.89
ES-allavg	0.722	70.33
SS-top	1.00	93.71
SS-3avg	0.664	28.88
SS-allavg	0.661	28.75
ES.SS-allavg	0.889	78.33

See Table 2 for aggregation abbreviations

#### Statistical models

**Fig. 3:**
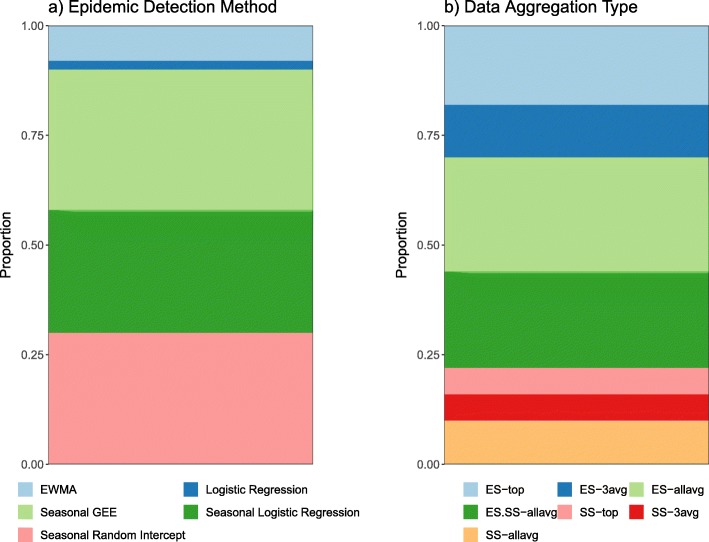
Characteristics of best-performing models. Representation of the proportion of **a**) model types and **b**) absenteeism data aggregation types within the 50 lowest FAR-producing epidemic detection methods. See Table 2 for aggregation abbreviations

The absenteeism data aggregations that resulted in the lowest FAR values for the model-based methods were those that incorporated the largest numbers of schools into their averages (ES-allavg and ES.SS-allavg). Of the ten models that produced the lowest FARs, all but two used one of these two absenteeism aggregations (Table 4). Even amongst the 50 lowest FAR-producing models, approximately half used absenteeism averaged over either all the schools or all elementary schools (Fig. 3). In particular, ES-allavg consistently produced results with low FARs. Table 5 shows the models that produced the lowest FAR for each absenteeism aggregation type and method of handling missing values; models that used ES-allavg absenteeism data had the lowest FARs regardless of whether deletion or interpolation in the training datasets was used. Secondary school absenteeism data was found to have lower predictive ability than elementary school data. None of the ten lowest FAR-producing model-based methods used secondary school absenteeism unless it was averaged together with elementary school data (Table 4).

**Table 4:** Epidemic detection methods with the ten lowest FARs

Model	Data Type	Parameters	FAR	ADD
Seasonal Mixed, D.O.W.	ES-allavg (Int.)	*l* = 7, *Θ* = 0.20	0.299	15.13
Seasonal Mixed	ES-allavg (Del.)	*l* = 11, *Θ* = 0.25	0.313	23.63
Seasonal Mixed, D.O.W.	ES-allavg (Del.)	*l* = 15, *Θ* = 0.25	0.333	21.50
Seasonal LR	ES-allavg (Del.)	*l* = 5, *Θ* = 0.25	0.344	23.00
Seasonal GEE	ES-top (Int.)	*l* = 1, *Θ* = 0.15	0.350	14.29
Seasonal GEE	ES-allavg (Del.)	*l* = 7, *Θ* = 0.25	0.350	23.13
Seasonal GEE	ES-3avg (Del.)	*l* = 15, *Θ* = 0.25	0.375	29.38
Seasonal LR	ES.SS-allavg (Del.)	*l* = 11, *Θ* = 0.30	0.375	31.75
Seasonal GEE, DOW	ES-allavg (Del.)	*l* = 11, *Θ* = 0.30	0.375	33.13
Seasonal GEE, DOW	ES.SS-allavg (Del.)	*l* = 8, *Θ* = 0.30	0.375	33.13

Del. = Missing values deleted, Int. = Missing values linearly interpolated

See Table 2 for aggregation abbreviations

**Table 5:** Best performing statistical models by data type, when missing values are either deleted or linearly interpolated

Data Type	Model	Parameters	FAR	ADD
**ES-top**				
Deleted	Seasonal Mixed, DOW	*l* = 7-8, *Θ* = 0.30	0.411	26.71
Interpolated	Seasonal GEE	*l* = 1, *Θ* = 0.15	0.350	14.29
**ES-3avg**				
Deleted	Seasonal GEE	*l* = 15, *Θ* = 0.25	0.375	29.38
Interpolated	Seasonal GEE	*l* = 6, *Θ* = 0.20	0.433	22.75
**ES-allavg**				
Deleted	Seasonal Mixed	*l* = 11, *Θ* = 0.25	0.313	23.63
Interpolated	Seasonal Mixed, DOW	*l* = 7, *Θ* = 0.20	**0.299**	**15.13**
**SS-top**				
Deleted	Seasonal Mixed	*l* = 4, *Θ* = 0.10	0.461	14.67
Interpolated	LR, DOW	*l* = 4, *Θ* = 0.25	0.454	9.17
**SS-3avg**				
Deleted	Seasonal GEE, DOW	*l* = 0, *Θ* = 0.25	0.420	21.00
Interpolated	Seasonal Mixed	*l* = 1, *Θ* = 0.15	0.422	21.57
**SS-allavg**				
Deleted	Seasonal GEE, DOW	*l* = 0, *Θ* = 0.25	0.420	21.43
Interpolated	Seasonal GEE, DOW	*l* = 0, *Θ* = 0.25	0.420	21.43
**ES.SS-allavg**				
Deleted	Seasonal LR	*l* = 11, *Θ* = 0.30	0.375	31.75
Interpolated	Seasonal LR	*l* = 4, *Θ* = 0.25	0.411	21.86

The metrics for the model with the lowest FAR are shown in bold. See Table 2 for aggregation abbreviations

Of the various modelling techniques that were considered for use as influenza epidemic detection methods, the EWMA models were the least represented amongst the best performing methods. None of the ten lowest FAR-producing epidemic detection methods used EWMA modelling, and even in the 50 best methods there were very few EWMA models. At best, the EWMA models were able to give an FAR of 0.438 and ADD of 32 days, these being obtained when the square-root transformed average of all elementary school absenteeism data with missing values deleted was used. Transforming the data by either taking the square root or log of absenteeism did not generally improve results compared to the untransformed data. Overall, EWMA models outperformed the 10% threshold method but were less successful than the regression-based models at achieving an acceptable balance between false and timely true alarms.

**Fig. 4:**
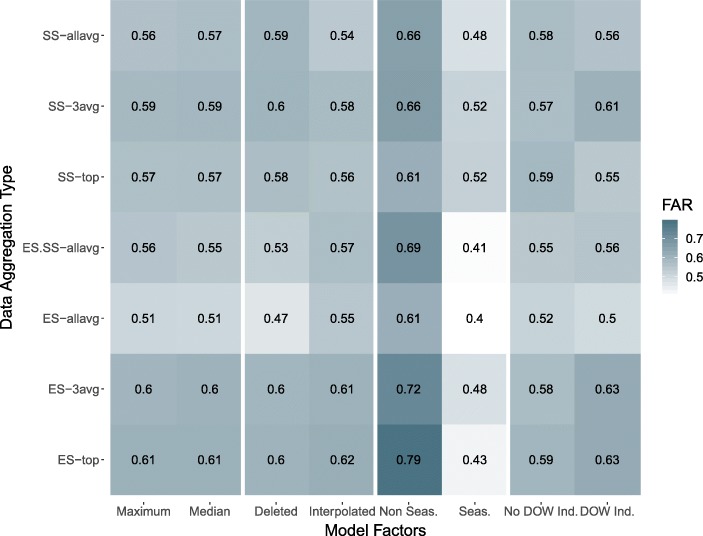
Characteristics of regression models. Effects of different model factors and absenteeism data aggregations on FAR averaged over all regression model types with optimized parameters. Each row represents a different aggregation for absenteeism data and each column represents either a data handling method or whether an additional predictor aside from absenteeism was included in the model. The pairs of columns separated by spaces can be compared to view the effect on FAR across the different data aggregations, where a lighter shade indicates preferable (lower) FAR. The value within each cell is the mean FAR

The best performing models were based on variations of the logistic regression model. Figure 4 summarizes the effect that the inclusion of various factors and different data aggregation types had on FAR across all the regression-based models. Seasonality terms seemed to have the greatest effect on the FAR, as models with these terms included tended to have considerably lower FARs than the non-seasonal models. Additionally, the ES-allavg data gave the most consistently low FARs with the regression-based models, with all other aggregation types performing variably well depending on what other factors were included in the model. Table 4 shows that, based on FAR, the ten best performing epidemic detection methods were all regression-based models with seasonality terms, all of which incorporated some form of elementary school absenteeism data. Between the mixed and GEE models, eight out of the ten best performing epidemic detection methods included a random intercept for school year. However, there was no clear pattern for day of week indicator or interpolated/ deleted missing values in the absenteeism training data. Linear interpolation of the missing absenteeism data in the training data for the model-based methods did not consistently improve the number of true alarms or reduce false alarms compared to when days missing values were simply deleted. Similarly, the inclusion of a categorical variable for day of the week improved FAR in some cases and worsened it in others.

**Fig. 5:**
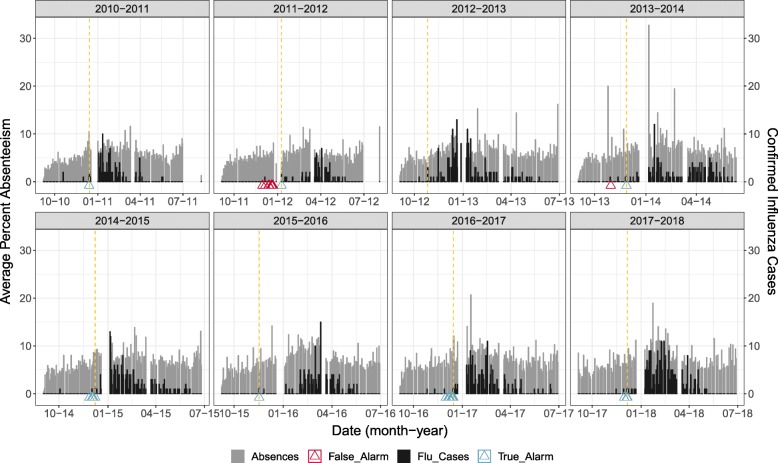
Alarms of the top-performing model. True and false alarms for the best performing model, faceted by school year: the seasonal logistic random intercept model using ES-allavg data with *l*=7, *Θ*=0.20, interpolated missing values in the training data. Averaged absenteeism is plotted as grey bars, with actual laboratory-confirmed influenza case counts overlaid as black bars, and the epidemic reference day is indicated by the dashed yellow line for each school year

The detection method with the lowest FAR was the seasonal mixed model with a day of week indicator, using ES-allavg absenteeism data with values linearly interpolated in the training sets. The optimized parameters were *l* = 7 days and *Θ*=0.20. Under this model, the start of each seasonal epidemic was captured with the exception of the 2012–2013 epidemic, and false alarms were only raised in two years. Figure 5 illustrates the timing of these alarms relative to the start of the influenza epidemic, where each panel represents a different school year where an alarm had the potential to be raised. True alarms tended to occur close to the start date of the seasonal epidemic. False alarms appeared to coincide with early cases of influenza that occurred far enough apart so as not to be classified as the start of the seasonal epidemic. Table 6 shows the number of false and true alarms, along with ADD, that were produced every school year in the study period when this model was used.

**Table 6:** Yearly alarms and ADD for the best performing model: the seasonal logistic random intercept model using ES-allavg data with *l*=7, *Θ*=0.20, interpolated missing values in the training data

School Year	False Alarms	True Alarms	ADD
2010–2011	0	1	13
2011–2012	8	1	14
2012–2013	0	0	55
2013–2014	1	1	14
2014–2015	0	4	3
2015–2016	0	1	14
2016–2017	0	8	0
2017–2018	0	3	8

Mean FAR = 0.299, mean ADD = 15.13

Parameter estimation was slower for models where missing absenteeism values in the training data had been linearly interpolated rather than deleted. The yearly results for the best model that used deletion are reported in Table 7. This model was also a seasonal mixed model using the ES-allavg absenteeism data, but did not include a day of week indicator. In comparison to the best epidemic detection method (the seasonal mixed model with day of week indicator and interpolated training data), this model captured one less epidemic giving it slightly a higher overall FAR, however it had fewer false alarms and the ADD was similar to the best performing model for the years where it did capture epidemics.

**Table 7:** Yearly alarms and ADD for the seasonal random intercept model with 11 lags and *Θ* = 0.25, using ES-allavg data with missing values deleted

School Year	False Alarms	True Alarms	ADD
2010–2011	0	2	13
2011–2012	0	1	14
2012–2013	0	0	55
2013–2014	1	1	12
2014–2015	0	2	7
2015–2016	0	0	70
2016–2017	0	5	5
2017–2018	0	1	13

Mean FAR = 0.313, mean ADD = 23.63

## Discussion

This paper proposed and tested several possible model-based epidemic detection methods as alternatives to the 10% absenteeism threshold method currently being used in WDG. For this study, an ideal model would be able to detect a seasonal epidemic earlier than it would be caught using reported laboratory-confirmed case counts alone, while not raising many false alarms. Overall, we found that all of the tested model-based approaches achieved these characteristics to a higher degree than the school absenteeism threshold method did.

The 10% threshold was able to produce true alarms with low ADD when it was applied to absenteeism averaged across all secondary schools, although this was counteracted by a high proportion of false alarms. This appears to contradict earlier findings that many schools would not reach 10% absenteeism even during an influenza season [15]. Secondary school absenteeism in the WDG region was higher than elementary school absenteeism in most school years, with an overall average absenteeism of 7.76% for the secondary schools that reported during the study period. A 10% cut-off may therefore be too low for secondary schools while being too high for elementary schools, and a more individualized approach such as that used in Mann et al. (2011) where thresholds are determined by standard deviations for a rolling mean may work better for WDG [16]. In WDG aggregated absenteeism data performed better within the 10% threshold method than using an individual school’s absenteeism for the purposes of detecting a regional epidemic. However, an alarm for an individual school may still be useful for detecting within-school outbreaks. Data do not currently exist for the evaluation of this use of the 10% threshold, and so this could be an area for future investigation.

The EWMA approach explored in this study is somewhat similar to the adaptive thresholds of Mann et al. (2005), although all past observations (as opposed to only 30) were included and weighted in the calculation of the standard deviation [16]. The EWMA models improved FAR compared to the threshold method but did not do as well as many of the regression-type models. Although data transformations were attempted, normality of the absenteeism data was not achieved, and the EWMA models may not have been able to sufficiently smooth out the day-to-day noise. Non-parametric modified EWMA models have been proposed for use with non-normally distributed data (see [40] for a review of several), however a challenge remains in choosing an appropriate subset of data to represent the “in-control” process; the distribution of absenteeism when influenza is not present in the community. In this study, the “in-control” mean was set to be the mean of all absenteeism data from the training years, however that average would include observations during an influenza season. Without knowing more information about additional circulating infectious diseases or other factors affecting absenteeism (which could be unique to different schools) it is difficult to choose a specific time period to use as the “in-control” process. In addition, most of the best-performing EWMA models had a very small optimized *λ* value, indicating that a high degree of smoothing was required. A small *λ* also approximates a CUSUM model, which has been used in previous studies where absenteeism is used for influenza surveillance [17, 18]. One such study concluded that the CUSUM was not ideal for their data [17]. Finally, at a small value of *λ* EWMA models can accumulate “credit” [41]. If an increase in mean absenteeism were to occur following a day where the EWMA statistic had been calculated to be a value less than *μ*_0_, it would take several days for the EWMA statistic to reach the UCL because of the low weights assigned to recent values, which may have limited the number of true alarms observed.

The regression-based models generally performed better than the EWMA models, particularly when covariates accounting for seasonality were included in the model. The seasonality (trigonometric) covariates may act to counterbalance any unusually high values of absenteeism that occur during times of the year when influenza is not generally circulating yet, thus reducing the number of false alarms. Inclusion of a random intercept for school year (in the mixed and GEE models) also seemed to improve model performance in most cases. The random intercept models were considered to allow for possible differences in the relationship between absenteeism and odds of an influenza case from year to year. However, to be able to estimate the random intercept, the models must be trained on at least some data from a given school year in order to be used to predict outcomes for that same year. In this study we used data from September of each year to obtain the intercept estimate and then began prediction in October. We tested models with up to 15 lagged school days of absenteeism, so this combined with the amount of data needed to estimate the intercept meant that in some years the models were not able to be used until nearly November. In the event of an unusually early seasonal epidemic starting prior to November, it would not be able to be captured by the random intercept models. Alternative solutions to estimating the random intercept for year-effect should be investigated. One possibility would be to use a previously estimated intercept for a new school year. For example, if information on the current influenza strain were available from earlier epidemics in a comparable nearby health region, an intercept could be used that was estimated on the WDG data from a previous year when there was a similar strain. Alternatively (and more realistically, given that WDGPH would not necessarily have access to strain information from other public health units), an intercept could be estimated using the most recent past year of WDG data and used with the current year’s data.

Secondary school absenteeism was less consistent than elementary absenteeism, and the yearly mean averaged across all secondary schools decreased over the 2012–2013 to 2015–2016 school years before increasing again (Fig. 1). This, along with the fact that fewer secondary schools than elementary schools report data, may explain why secondary school absenteeism generally performed less well than elementary school absenteeism in predicting influenza epidemics. Despite this, an Augmented-Dickey-Fuller test for non-stationarity found all absenteeism aggregations to be stationary, suggesting that the absenteeism decrease was not enough to cause a significant change in temporal trend of the data. Preliminary analysis based only on the 2008–2014 data yielded better performance of the models using the secondary school data than was seen using the full dataset. If the predictive abilities of secondary school absenteeism have decreased in more recent years, it may not be worth continuing to collect secondary school absenteeism data.

Absenteeism data is voluntarily reported to WDGPH daily by schools through an on-line system. The current system (introduced in 2017) features a field where school administrators can choose to enter the number of absences related to illness (in total, as well as categorized as respiratory, gastrointestinal and other). The illness and specific syndrome counts were observed to be unreliable, (for example exceeding the total number reported absent), and incomplete and thus could not be used in this study. Increased adoption of this reporting feature by school administrators could greatly aid model performance as observations from schools that are experiencing an outbreak or epidemic of an illness with non-respiratory symptoms could be excluded. Reporting of other reasons for unusual levels of absenteeism, such as field trips, would be similarly helpful. However, even if more detailed absenteeism information were available, a school-absenteeism surveillance system has limitations due to the “missing” data from weekends and holidays as well as potentially differing effects of different influenza strains on children. For example, Cauchemez et al. (2008) found that influenza subtype B was more closely associated with children than subtype A\H3N2 [42]. One way to overcome these limitations would be to use a second surveillance system in addition to the absenteeism surveillance system. Possibilities could include an alternative form of syndromic surveillance that targets an older demographic (such as over-the-counter drug sales monitoring).

We chose to define the start of an influenza epidemic by the first time two cases were reported to WDGPH within a week of each other. In the WDGPH region, there are generally no laboratory-confirmed influenza cases observed outside of influenza season, however if influenza activity became more sustained throughout the year in the region, or in a region with a larger population where more cases are seen overall, it may be necessary to explore alternate definitions for the beginning of the epidemic. The Moving Epidemic Method could be used to identify an influenza activity threshold above which the region would be defined as experiencing an epidemic [43].

Another limitation of this study is that spatial information was not incorporated in any of the model-based epidemic detection methods. Approximately half of the WDG population live in rural areas, which accounts for 98% of the geographic area serviced by WDGPH [21, 22]. Since spatial identifiers were not provided with the absenteeism data, we were unable to select the top or top three consistently reporting schools to be representative of a certain part of the region (ie. the City of Guelph) and so they were selected on the basis of most days reported. Therefore, some or all of the top schools chosen here could be in relatively remote locations. Depending on where the annual influenza season begins within the region in any particular year, influenza cases in Dufferin may precede cases in Guelph for example, resulting in absenteeism in a Guelph school raising an alarm too late for WDG as a whole. Since WDGPH has access to school identifiers, it may be more useful for them to choose a few well-reporting schools from each of the larger towns in the region and run the chosen epidemic detection method on the separate locational averages in parallel.

Another area for future work is the development of an improved metric or collection of metrics to use for epidemic detection method evaluation. We chose to optimize model parameters based on the lowest FAR since selecting based on the ADD would lead to too many false alarms (false alarms increase somewhat proportionately with true alarms). The FAR provides an idea of the balance between true and false alarms, but does not indicate where these alarms occur relative to the start of an epidemic. A low FAR can be misleading in certain cases; for example, a method that raised no false alarms and a single true alarm on the reference day each year would have a FAR of 0 when in reality the method is not giving an alarm any sooner than one that would be raised based on hospital reports alone. An improved metric for identifying optimal model parameters might define an ideal day (for example, 14 days prior to the reference day) for an alarm to be raised, and penalize both false and true alarms based on the difference between the day on which they were raised and the ideal alarm day. This would also penalize models that raise alarms clearly unrelated to influenza activity more heavily than those whose alarm dates are only marginally outside the true alarm range.

When using any of these epidemic detection methods in practice, it would be useful to test model performance as more years of data become available, and obtain updated optimized parameters on a yearly basis. Since linear interpolation increases computation time for training models and was not found to significantly improve the results of methods compared to when the missing values were deleted, deleting missing values is recommended. The number of lagged days of absenteeism data included as predictors in the model and the probability thresholds for the regression-based models reported in this paper are optimized for the data currently available, but should be re-evaluated regularly to maintain the efficacy of influenza epidemic detection methods of this nature. The methods identified in this study should also be evaluated with data from other public health regions prior to being used as epidemic detection method outside of WDG. Regional differences in weather, school attendance patterns, health care, and other factors could influence which method would be the most effective.

## Conclusions

Comparison of the 10% threshold approach to several modelling-based methods showed that the 10% threshold approach can be improved upon to reduce the number of false alarms while still giving warning of influenza epidemics ahead of laboratory-confirmed cases. Based on our findings, a seasonal logistic regression model with random intercept for school year is recommended for influenza surveillance in WDG. This approach was able to raise true alarms for almost every epidemic of influenza. Although it produced some false alarms, those alarms did coincide with influenza cases, though those cases were spread too far apart to be classified within an epidemic. In practice these alarms may still be useful in alerting WDGPH of when influenza or a similarly transmitted disease begins circulating in the region. Absenteeism averaged over all reporting elementary schools is recommended as the model predictors, as it was found to give the best balance of true and false alarms for most of the epidemic detection methods. However, it is suggested that the school absenteeism surveillance system be used in conjunction with another influenza surveillance system, due to limitations caused by missing data and absenteeism patterns unrelated to influenza.

## Data Availability

Under a data sharing agreement, the data for this study were made available by, and used with permission from, Wellington-Dufferin-Guelph Public Health. Access to, and permission to use, the data must be obtained from Wellington-Dufferin-Guelph Public Health, Jennifer MacLeod, Manager, Health Analytics (jennifer.macleod@wdgpublichealth.ca ).

## Abbreviations

ADD: Accumulated days delay
CUSUM: Cumulative sum
DOW: Day of the week
ES: Elementary school
EWMA: Exponentially weighted moving average
FAR: False alarm rate
GEE: Generalized estimating equation
SS: Secondary school
UCL: Upper control limit
WDG: Wellington-Dufferin-Guelph
WDGPH: Wellington-Dufferin-Guelph Public Health
